# The Sexual Self as a Function of Relationship Status in an Emerging Adult Sample

**DOI:** 10.3390/bs13060505

**Published:** 2023-06-16

**Authors:** B. J. Rye

**Affiliations:** Department of Psychology, St. Jerome’s University at the University of Waterloo, 290 Westmount Road North, Waterloo, ON N2L 3G3, Canada; bjrye@uwaterloo.ca; Tel.: +1-519-884-8111 (ext. 28219)

**Keywords:** sexual selfhood, sexual self, sexual self-concept, erotophobia–erotophilia, sexual comfort, relationship status

## Abstract

A sample of emerging adult university students completed a survey with the goal of investigating components of “the sexual self” and how these constructs were influenced by romantic relationship status. Three general aspects of the sexual self were considered: sexual self-concept, comfort with sexuality, and past sexual behavior. Sexual self-concept was defined as composed of constructs such as sexual self-schema, self-efficacy, consciousness, optimism, problem self-blame, power/other control, and motivation to avoid risky sex. Sexual comfort, conceptualized as a personality disposition of erotophobia–erotophilia, was assessed using three instruments. This included the Sexual Opinion Survey, the original individual difference measure of erotophobia–erotophilia. Past sexual behavior was assessed with the Derogatis Sexual Functioning Inventory. Findings suggested that individuals in a relationship had more positive sexual self-concepts and were more erotophilic and comfortable with sexuality generally. These differences were modest, based on effect size statistics. Past sexual experience also differed, contingent on relationship status. Some sexual self-concept scales were predictive of sexual satisfaction, while comfort with sexuality was predictive of relational satisfaction. Romantic relationships may have important implications for sexual selfhood, but this is a tentative suggestion, as this was a correlational study and the relationships are likely bidirectional.

## 1. Introduction

The self is the core of cognition, affect, and behavior in psychology [1]. Self is defined by identities, comprised of components such as characteristics, traits, roles, and group membership, and is informed by self-knowledge. Self-concept is the term used to describe this self-knowledge, which is constructed through cognition [2,3]. The self-concept is a multidimensional theoretical construct thought to have many dimensions; for example, self-esteem, academic self-concept, and global self-concept have all been investigated from a self-concept lens [4].

Theorists have recognized the critical role of social relationships in the construction of self-concept; our definition of ourselves often has to do with how we relate to other people and how integrated and accepted we feel by significant others [3]. According to Feiring and Taska [5], we are born into a social situation that becomes the basis of our self-concept; this first involves nuclear family relationships (e.g., caretakers, siblings) and progresses to include teachers, friends, peers, and the like [6]. This social network then typically continues to develop so as to include romantic partners. These close relationships are a defining, critical component of who we are as human beings—to the extent that “me” often becomes “we”. That is, our identity often involves integrating a beloved one into one’s self [7]. Tice and Baumeister [8] argue that the self-concept does not merely develop as self-knowledge from our interpersonal interactions. Rather, they state that “[r]elating to others is part of what the self is for” (p. 71), meaning that much of the function of the self-concept is to foster interpersonal relationships. This study investigates a particular aspect of the self—the sexual self—pertaining to romantic relationships.

### 1.1. The Sexual Self

In terms of sexuality, one area that has proliferated is the discussion of sexual identity, which has often referred to sexual orientation [9], gender-role-related self-schemas [10], or even biological sex [11]. Worthington et al. [12] attempted to conceptualize sexual identity in a multidimensional and developmental manner. However, their model focused mainly on sexual activities and has been applied primarily to the examination of sexual orientation [9,13,14]. What is often ignored is sexual self or selfhood; how one conceives of the sexual self. Models such as Worthington et al.’s [12,13,14] include little about one’s sexual self-concept. Sexual self-concept takes into consideration a multitude of personal cognitive aspects of sexuality (e.g., sexual self-schemas, sexual motivation) and has been described as the core of one’s sexual self [15]. Therefore, sexual self-concept is used as an important conceptual indicator of the sexual self in the current study.

Sexual self-concept is a multidimensional construct that refers to the perceptions and feelings an individual has about themselves as a sexual being [15,16]. The development of this dimension is an important task of adolescence [17], with changes during this time following a trajectory of increased comfort with sexuality and sexual behaviors, and lower levels of sexual anxiety and avoidance [18]. Some scholars posit that changes in self-concept are a result of experiences, with confidence increasing after participation in sexual activities [19,20]. Others discuss sexual self-concept domains (e.g., sexual self-efficacy) as being predictive of safer sexual behavior [21]. Using a longitudinal design to illustrate the dynamic and reciprocal nature of the relationships, Hensel et al. [18] found that changes in sexual self-concept were predictive of and responsive to sexual behavior. Regardless of potential bidirectionality, it is clear that an individual’s sexual self-concept as well as sexual behavior are important in conceptualizing the self.

While self-concept is generally thought of as beliefs, thoughts, or cognitive representations of oneself, some studies of sexual self-concept involve affective domains, such as sexual anxiety [18] and sexual depression [22]. However, these are very specific as they relate to affective assessments of one’s sexual life (e.g., “I feel anxious when I think about the sexual aspects of my life”). In contrast, a broader affective approach involves erotophobia–erotophilia, which is a learned avoidance–approach response to sexual stimuli and has been characterized as a dimension of personality [23]. This affective response includes personal sexual experiences (e.g., “Seeing an erotic movie would be sexually arousing to me”), but goes further to include one’s affective responses to or comfort with other people’s sexuality, which can be reflected in attitudes toward sexuality vis à vis others (e.g., “It would be emotionally upsetting for me to see someone exposing themselves publicly”). Thus, including general comfort with sexuality is a different type of affective component of the sexual self. Erotophobia–erotophilia is not necessarily part of our cognitive self-perception, but one’s comfort with sexuality may help guide one’s sexual interactions and behavior. Sexual self-concept and erotophobia–erotophilia are conceptually related [24]. While influenced by experience, our sense of our sexual self—comprising self-concept and affective responses—serves to help us regulate our behavior.

### 1.2. Relationships and Sexual Selfhood

As one of the core principles in relationship research, Finkel et al. [7] identified that two partners merging into a single psychological entity is more than merely the sum of the two partners. They call this phenomenon integration and discuss how this interdependence within a close relationship results in the blurring of personal boundaries such that the “me” becomes a “we”. This merging is so potent that partners sometimes have trouble distinguishing between traits [25] and behaviors [26] of themselves and their romantic partner. This interdependence and integration may also be characterized as a self-expansion feature of love.

There has been considerable theorizing about and research into the effects of close, romantic relationships on self-identity construction. For example, Aron, Ketay, Riela, and Aron [27] suggest that the incorporation of an intimate other into one’s self-identity influences feelings about the self, such as self-esteem. This body of literature is largely silent, however, on the impact that close relationships have on sexual components of identity; for example, sexuality is rarely mentioned in volumes on the self and close relationships [28,29,30]. Since romantic relationships typically involve sexual components, it is logical that being in such a relationship would affect the sexual self-conception.

Sexual self-schemas are an aspect of sexual self-concept that have been examined for those in relationships. Having a positive sexual self-schema has been shown to increase one’s perception of partner satisfaction, in turn benefiting one’s own satisfaction [31,32]. Additionally, compared to those with negative sexual schemas, individuals with positive schemas have been found to have a larger number of sexual partners and a wider range of sexual experiences [33]. It appears that positive self-schemas and self-efficacy, which are dimensions of sexual self-concept, lead to increased perceptions and feelings of sexual satisfaction for those in romantic relationships. As increased sexual satisfaction has been found to promote relationship stability and quality [34], we can theorize that self-concept is influenced by relationship status.

A few studies have investigated romantic relationship status and some self-related measures. In a longitudinal study of the dating relationship trajectories of an adolescent sample, Davies and Windle [35] found that being in a romantic relationship was associated with some indicators of positive emotional adjustment—such as enhanced self-rating of attractiveness and decline in depressive symptoms—relative to those not in relationships. Being in a relationship was also associated with increased sexual activity. Sexual satisfaction has also been found to be a function of relationship status with those in relationships more satisfied with their sex lives [22,36,37].

### 1.3. Research Question

The general research question of the current study is whether people differ in how they characterize themselves sexually as a function of the type of romantic relationship in which they are currently. A couple of studies somewhat addressed this question [22,36], but mainly focused on sexual satisfaction as the outcome. In the current study, sexual self-characterization or sexual selfhood was conceptualized broadly as sexual self-concept (i.e., sexual self-schema, consciousness, self-efficacy, optimism, problem self-blame, power/other sexual control, and motivation to avoid risky sex), comfort with sexuality (i.e., comfort with sexual behavior, erotophobia–erotophilia, and attitudes toward sexual curiosity in children), and past sexual behavior. Relationship status was categorized in an ordinal fashion (i.e., unattached or in a casual or a serious romantic relationship) and used as a grouping or quasi-independent variable. Specifically, those who were involved in romantic relationships were hypothesized to have more favorable or well-defined sexual self-concept, greater comfort with sexuality, and a more extensive sexual behavior history.

While focusing on sexual selfhood and relationship status primarily, this study addresses the relationship of the sexual self and satisfaction with sexual functioning. By extension, sexual identity or selfhood—sexual self-concept, sexual comfort, and sexual behavior—was investigated as predicting relationship satisfaction given that relationship satisfaction is linked with sexual satisfaction [34,38].

## 2. Materials and Methods

### 2.1. Participants

Participants were 183 first-year university students (*n*_women_ = 117, *n*_men_ = 63, *n*_unknown_ = 3; $\overset{-}{x}$_age_ = 18.39, sd = 1.14, range 16–24 years; 46% Caucasian and 39% Asian/South Asian) who participated for course credit or CAD $7 remuneration. Just under 60% were students enrolled in the Faculty of Arts, while 32% were enrolled in Science or Health Sciences Faculties. Most participants (*n* = 177) provided complete data; participants were included in analyses in a pairwise fashion where possible.

### 2.2. Instruments and Measures

#### 2.2.1. Relationship Status

Participants indicated whether they were currently involved in a romantic relationship; if yes, for how long. They were asked to choose the type of relationship from a list of options, which included a free-response option to describe relationship type. Two coders assigned a relationship category based on these relationship items. Consequently, participants were coded as not being in a relationship (single, *n* = 90, 50% of the sample), being in a casual relationship (less serious, *n* = 38, 21% of the sample), or being in a serious relationship (more serious, *n* = 54, 30% of the sample). One participant’s relationship status could not be classified.

#### 2.2.2. Satisfaction

Two satisfaction items were included in the survey. One assessed satisfaction with sexual functioning and the other with relationship satisfaction. These were included under a background information section and preceded the other instruments in the survey. Both items (“How satisfied are you with your current relationship?” and “How satisfied are you with your sexual functioning?”) had 7-point response scales ranging from extremely dissatisfied to extremely satisfied.

#### 2.2.3. Sexual Self-Concept

Sexual self-concept was measured using seven scales from Snell and Kilimnik’s Multidimensional Sexual Self-Concept Questionnaire [39]. Sexual self-schema is how one thinks, cognitively, about oneself sexually (e.g., “Not only would I be a good sexual partner, but it is important to me that I be a good sexual partner”). Sexual self-efficacy is one’s self-assessment of one’s ability, competency, skills, and the like regarding one’s own sexual needs (e.g., “I have the ability to take care of any sexual needs and desires that I might have”). Sexual consciousness involves being in tune with or reflective on sexual aspects of the self, such as sexual desires, thoughts, and ideas (e.g., “I am very aware of my sexual feelings and needs”). Sexual optimism is the endorsement of positive beliefs regarding one’s future sexuality (e.g., “I expect that the sexual aspects of my life will be positive and rewarding”). Sexual problem self-blame encompasses self-attributions of blame or personal responsibility for negative sexual outcomes such as sexual health or dysfunction (e.g., “I would be to blame if the sexual aspects of my life were not going very well”). Power/other sexual control is a form of external locus of control or learned helplessness—the belief that powerful others control one’s sexuality (“My sexual behaviors are determined largely by other more powerful and influential people”). Motivation to avoid risky sex is the desire to refrain from unhealthy sexual behaviors (e.g., “I am motivated to avoid engaging in risky sexual behavior”).

Each scale was composed of five statements to which responses were made on a 5-point “characteristic of me” scale (1 = not at all, 2 = slightly, 3 = somewhat, 4 = moderately, 5 = very). Averaging scores on the individual items created a composite score for each sexual self-concept scale (after appropriate reverse coding). The sexual self-concept scales have sufficient scale score reliabilities; Snell and Kilimnik [39] reported an alpha range from 0.72 (Motivation to avoid risky sex) to 0.87 (Sexual self-schema) with the Cronbach’s alphas in the current study, similar to Snell and Kilimnik—falling between α = 0.64; ω = 0.56 (Sexual optimism) and α = 0.89; ω = 0.89 (Sexual efficacy). Snell and Kilimnik also presented an overview of validity information for these scales.

#### 2.2.4. Comfort with Sexuality

Comfort with sexuality was measured using Comfort with Sexual Behavior [40] and the Sexual Opinion Survey, the original measure of erotophobia–erotophilia [41], as well as Attitudes toward Sexual Curiosity in Children [40] scales.

##### Comfort with Sexual Behavior

Adapted from Zuckerman’s Attitudes toward Heterosexual Activities scale [40], participants indicated their opinion on what they felt was an acceptable behavior for most persons their age and gender on a 10-item questionnaire surrounding specific sexual behavior (e.g., “Erotic kissing” and “Sexual intercourse, face-to-face position”). Responses were couched in relational contexts using a 6-point acceptability scale (options included: (1) Never all right regardless of how much you love the person, (2) All right if you are married, (3) All right if you are engaged/live together, (4) All right if you are deeply in love with/dating seriously, (5) All right if you have strong affection/dating casually, and (6) All right regardless of how you generally feel about the person or how long you have known them). Averaging individual item responses produced a composite score; higher scores represented more liberal relational attitudes. Scale score reliability for the current study was quite high (Cronbach’s α = 0.93; ω = 0.92) and similar to that of the original instrument (Cronbach’s α = 0.91) [40].

##### Erotophobia–Erotophilia

The Sexual Opinion Survey (SOS) is the standard measure of erotophobia–erotophilia, a personality dimension described as the learned disposition to respond to sexual stimuli with affect and evaluations that range from negative (erotophobic) to positive (erotophilic) [23,41]. The dimension of erotophobia–erotophilia is linked with a variety of behaviors that reflect broad-based avoidance or approach to sexual stimuli. Each item discusses a subject of a sexual nature (e.g., “The thought of engaging in unusual sex practices is highly arousing”) and participants indicated their responses on 7-point Likert—Strongly Disagree to Strongly Agree—scales. A composite score was computed by averaging the responses on 21 individual items (after appropriate reverse coding so higher scores represent greater erotophilia). The SOS has been reported to have well-established validity and high scale score reliability (Cronbach’s α = 0.76 to 0.89) [41]; in the current study, reliability was quite high (Cronbach’s α = 0.91; ω = 0.87).

##### Attitudes toward Sexual Curiosity in Children

Participants indicated their agreement on a 7-point scale with 12 belief statements about harm caused by sex, normalcy of interest in sex, or withholding knowledge about sex vis à vis children (e.g., “There is usually something wrong with a child who asks a lot of questions about sex” and “Sex play is a normal thing in children”) [40]. Averaging individual items responses produced an aggregate score; after appropriate reverse coding, higher scores indicated greater comfort with children’s curiosity toward sex. Reliability for the current study was adequate (Cronbach’s α = 0.76; ω = 0.76). Zuckerman did not report Cronbach’s alpha, but presented test–retest reliability scores between 0.44 and 0.64; validity information was also presented [40].

#### 2.2.5. Past Sexual Behaviors

Finally, past sexual behaviors were assessed using an adaptation of the Experience subtest of the Derogatis Sexual Functioning Inventory [42], along with a variety of individual items (e.g., age at first intercourse, number of lifetime sexual partners) related to sexual activity. The original Sexual Functioning Inventory was reported to have good psychometric properties [43]. Using cluster analysis, participants were classified into one of three past sexual behavior groups: sexually inexperienced, having engaged in very few sexual behaviors; somewhat experienced, having engaged in some sexual genital sexual behaviors with others but usually not intercourse; or experienced, representing those who had participated in many sexual behaviors and usually included intercourse.

### 2.3. Procedure

Participants, solicited from first-year undergraduate classes, voluntarily completed a paper-and-pencil questionnaire either in an empty university room or at a location of their own choice. The questionnaire included demographic items first. Embedded within a larger survey, the sexual self-concept, sexual comfort, and sexual behavior instruments, respectively, were presented at the end of the survey. Upon returning the questionnaire, participants were thanked, remunerated, and given a debriefing information sheet. Procedures were in accordance with Canadian Psychological Association guidelines and approved by the university’s institutional review board.

### 2.4. Analysis Plan

First, statistics describing the dependent variables (i.e., sexual self-concept, comfort with sexuality, and sexual behavior) were calculated. Additionally, how instruments assessing each of these concepts interrelated was presented. Addressing the key research question, whether the sexual self differs as a function of relationship status, multivariate analyses of variance were conducted for sexual self-concept, comfort with sexuality, and past sexual behavior, with concurrent univariate analysis of each distinct scale, compared based on relationship status. Discriminant function analyses were conducted to investigate the underpinnings of the MANOVAs, as well. Finally, sexual self-concept, comfort with sexuality, and past sexual behavior as predictors of relationship and sexual functioning satisfaction were explored using bivariate correlations and multiple regression analyses. Analyses were conducted using SPSS v.29.

## 3. Results

### 3.1. Descriptive Statistics

Participants had a positive sexual self-concept generally (see Table 1). On average, the participants were highly motivated to avoid risky sex and they were reflective or conscious about their sexuality. They also had a well-defined cognitive structure about themselves (i.e., positive sexual self-schema); were sexually optimistic; and felt they had agency and competency regarding their own sexuality (i.e., sexual self-efficacy). Further, participants did not overly blame themselves for sexual problems (i.e., although the average indicates “somewhat characteristic” of the self). Others with control over the sexual aspects of one’s life received an average rating of “slightly characteristic of me”. In essence, participants generally denied an external locus of control regarding their sexuality; this suggests they believed themselves to be more in control of the sexual aspects of their lives relative to powerful others. Overall, the distribution of scores tended to be skewed toward a favorable sexual self-concept.

Participants were slightly comfortable with sexuality, in general. Participants generally endorsed sexual behaviors as acceptable as long as they occurred within a loving, dating relationship. On average, participants were slightly erotophilic. Participants were slightly comfortable with the sexual curiosity of children ($\overset{-}{x}$ = 4.24, sd = 0.81; where a score of 7 indicated the greatest comfort/liberalism with children’s curiosity about sexuality). All three comfort with sexuality variables were approximately normally distributed.

While not everyone completed the sexual experience inventory, of those who did, 32% were classified as inexperienced (*n* = 48), 26% were classified as somewhat experienced (*n* = 39), and 42% were classified as experienced (*n* = 64). In terms of the individual sexual behavior items, the average age of first intercourse was 16.5 years (standard deviation = 1.8 years; median and mode = 16 years). Of those who provided responses, 32% reported having zero other-sex partners, 24% reported one other-sex partner, and 43% reported having had two or more other-sex partners, while 87% reported zero same-sex partners. Three quarters reported having no one-night stands.

The dependent variables (i.e., sexual self-concept, comfort with sexuality, past sexual behavior) and the grouping variable were examined for gender differences. There were no gender differences except for sexual problem self-blame where men characterized themselves as more blameworthy than women did ($\overset{-}{x}$_men_ = 3.52, sd = 0.92 vs. $\overset{-}{x}$_women_ = 2.95, sd = 0.92; *t*(175) = 3.96, *p* < 0.001, Cohen’s *d* = 0.92).

### 3.2. Correlations among the Sexuality Self Variables

The sexual self-concept scales were moderately related to each other with an average intercorrelation of 0.34 (*p* < 0.0001; Fisher’s r-to-z). Self-schema, self-efficacy, and consciousness were strongly correlated. The three comfort with sexuality dependent variables (average correlation = 0.55, *p* < 0.0001; the average correlations were calculated using Fisher’s r-to-z transformation) were moderately-to-strongly intercorrelated [44]. Table 1 presents bivariate correlations.

The four measures of past sexual behavior (number of same- and other-sex partners, number of one-night stands, and the sexual experience category) demonstrated a relatively wide range of relationships. These past sexual behavior variables intercorrelated *r* = 0.34, *p* < 0.0001. Low correlations were mainly a function of number of same-sex partners not correlating well with the other sexual behavior measures. Most notably, there was no relationship between number of same-sex partners and the three-level sexual behavior experience classification variable. Thus, the sexual behavior experience variable is likely reflective of heterosexual sexual behavior.

### 3.3. Sexual Selfhood Differences as a Function of Relationship Status

#### 3.3.1. Sexual Self-Concept

Multivariate Analysis of Variance indicated differences in sexual self-concept as a function of relationship status (Pillai’s Trace *V* = 0.25, Multivariate *F*(14, 338) = 3.40, *p* < 0.0001; η_p_^2^ = 0.12; underlying multivariate normality assumptions were met: Box *M* = 74.23, *F*(56, 42337.19) = 1.24, *p* = 0.11). Univariate tests (Table 2) indicated that, generally, people in relationships had a more favorable sexual self-concept compared to those who were single, as evidenced by modestly strong significant effects for sexual self-schema, self-efficacy, consciousness, and optimism.

The MANOVA analyses were explored further using discriminant function analysis to investigate the linear combination of sexual self variables relative to the relationship status variable. The discriminant function analysis for sexual self-concept revealed two discriminant functions. Function 1 explained 80.4% of the variance, canonical *R*^2^ = 0.19, and the second function explained 19.6% of the variance, canonical *R*^2^ = 0.06. The first discriminant function (i.e., from function 1 to 2) differentiated the relationship status groups (Wilks’ Λ = 0.76, χ^2^(14) = 46.12, *p* < 0.0001). After the first function was taken into account, the second function was non-significant in terms of differentiating between relationship status groups (Wilks’ Λ = 0.95, χ^2^(6) = 9.62, *p* = 0.14). Correlation of the sexual self-concept variables and the discriminant functions (Table 3) indicated that sexual self-schema, sexual self-efficacy, and sexual consciousness loaded more highly on the first function relative to the second function. Sexual problem self-blame and power/other sexual control loaded weakly and negatively on the first function and even more weakly yet positively on the second function. Sexual optimism and motivation to avoid risky sex loaded more highly on the non-significant second function than the first. The discriminant function plot suggested that the first variate distinguished between those not currently in a relationship and those in any relationship.

#### 3.3.2. Comfort with Sexuality

There was a trend in those in relationships to be more comfortable with sexuality than those who were not in relationships. However, this statement is made with strong caution as the multivariate test was—at best—only marginally significant and the effect was extremely weak (Pillai’s Trace *V* = 0.07, Multivariate *F*(6, 346) = 2.04, *p* = 0.06; η_p_^2^ = 0.03; underlying multivariate normality assumptions were met: Box *M* = 14.98, *F*(12, 64318.95) = 1.21, *p* = 0.27). Table 2 presents the descriptive and univariate statistics associated with these tests.

The follow-up discriminant function analysis for comfort with sexuality produced two non-significant discriminant functions. Function 1 explained 91.4% of the variance, canonical *R*^2^ = 0.06, and the second function only explained 8.6% of the variance, canonical *R*^2^ = 0.01. The first discriminant function marginally differentiated relationship status groups (from function 1 to 2; Wilks’ Λ = 0.93, *F*(6) = 12.15, *p* = 0.059; function 2 was non-significant Wilks’ Λ = 0.99, *F*(2) = 1.08, *p* = 0.58). Correlations of comfort with sexuality variables and the discriminant functions (Table 3) indicated that all of the sexual comfort variables contributed to the first function, although attitudes toward the sexual curiosity of children was of lesser importance than the other two. For the second, non-significant function—after the variance because the first variate was taken into account—sexual curiosity in children was most important, followed by comfort with sexual behavior. The first function seemed to represent sexual comfort in relation to the self, whereas the remaining variance may have represented feelings about the sexuality of others. The inspection of the group centroids suggests that the first function distinguished between those not currently in a relationship and those who were in a relationship (either more or less serious). However, it is important to qualify these interpretations by the fact that neither function was significant and both demonstrated very small canonical correlations with relationship status groupings.

MANCOVA was considered for sexual self-concept and comfort with sexuality dependent variables with virginity status as a covariate. However, the covariate—ever had intercourse—was correlated with the grouping variable of relationship status (*r* = 0.51). Thus, the requirement of independence of the treatment variable and the covariate was not met [45], and these tests were rejected.

#### 3.3.3. Past Sexual Behavior

There was a multivariate difference in sexual behavior as a function of relationship type (Pillai’s Trace *V* = 0.26, Multivariate *F*(8, 282) = 5.26, *p* < 0.0001; η_p_^2^ = 0.13; underlying multivariate normality assumptions were not met: Box *M* = 116.88 *F*(20, 29050.38) = 5.56, *p* < 0.001). The inspection of Levene’s test of equality of error variance indicated that only the sexual experience variable demonstrated unequal variance, likely a result of the mid-sized group having lower variance (i.e., not a serious violation [45]). The univariate effects (Table 2) indicated that those in more serious relationships were more sexually experienced relative to those in less serious relationships, who were, in turn, more sexually experienced than single individuals based on the three-group experience variable. Those in less serious relationships had more partners than those who were single. No other partnership differences were found (i.e., one-night stands, number of same-sex partners).

The past sexual behavior discriminant function analysis produced two discriminant functions. Function 1 explained 94.2% of the variance, canonical *R*^2^ = 0.24, and the second function only explained 5.8% of the variance, canonical *R*^2^ = 0.02. The first discriminant function differentiated relationship status grouping (From function 1 to 2; Wilks’ Λ = 0.75, *F*(8) = 41.68, *p* < 0.001; function 2 was not significant Wilks’ Λ = 0.98, *F*(3) = 2.74, *p* = 0.43). The correlation of past sexual behavior variables and the discriminant functions (Table 3) indicated that the sexual experience variable contributed extremely strongly to this first function, followed by number of other-sex partners. The second, non-significant function was driven by number of other-sex partners followed by number of one-night stands and number of same-sex partners. The first function seemed to be about heterosexual sexual behavior, while the second non-significant function may be described as more sociosexual orientation. Inspection of the group centroid plot suggested that the first variate distinguished between those not currently in a relationship and those in a more serious relationship; those in a less serious relationship were in between the two.

### 3.4. Relationship Status, Sexual Selfhood, and Satisfaction

An analysis of variance assessed whether satisfaction with sexual functioning differed contingent upon relationship status. This revealed a significant but modest effect of relationship status on sexual functioning satisfaction (Welch’s *F*(2, 88.63) = 4.95, *p* = 0.009, η_p_^2^ = 0.06; Levene’s test *F*(2, 165)_median_ = 2.92, *p* = 0.057 [45]). Those in a more serious relationship ($\overset{-}{x}$_more serious_ = 5.89, sd = 1.16, *n* = 53) were significantly more satisfied with their sexual functioning relative to those who were single ($\overset{-}{x}$_single_ = 5.23, sd = 1.22, *n* = 79). Those who were in less serious relationships ($\overset{-}{x}$_less serious_ = 5.58, sd = 1.20, *n* = 36) did not differ from those who were in more serious relationships or those who were single, determined using Student–Newman–Keuls post hoc analysis.

In terms of relationship satisfaction, those in a more serious relationship ($\overset{-}{x}$_more serious_ = 6.40, sd = 0.79, *n* = 53) were compared to those in a less serious relationship ($\overset{-}{x}$_less serious_ = 4.97, sd = 1.64, *n* = 34). Those who were in a more serious relationship expressed significantly more relationship satisfaction (adjusted *t*(43) = −4.72, *p* < 0.001, Cohen’s *d* = 1.20).

#### 3.4.1. Correlations of Satisfaction with Sexual Functioning

Table 4 presents the correlations of sexual functioning satisfaction with sexual self-concept and comfort with sexuality variables as well as past sexual behavior. These are presented separately by relationship status. Partial correlations between satisfaction with sexual functioning and the sexual selfhood variables for all participants controlling for relationship status are presented, too. Overall, these relationships were weak, but there was a general pattern that satisfaction with sexual functioning and sexual self-concept variables were modestly correlated for those in a serious relationship (average *r* = 0.33; calculated using absolute valence and Fisher’s r-to-z-transformation) or single (average *r* = 0.25), but not for those who were in a less serious relationship (average *r* = 0.15). Correlations between satisfaction with sexual functioning and comfort with sexuality as well as with past sexual behavior were generally weak and not significant.

Further, a multiple regression analysis was conducted with sexual satisfaction as the dependent variable and the sexual self-concept variables, comfort with sexuality variables, the sexual experience variable, as well as relationship status (i.e., more serious, less serious, or single) as predictor variables. The equation was significant (*F*(12, 120) = 4.05, *p* < 0.001, *R* = 0.54), where sexual consciousness (β = 0.33), sexual self-schema (β = 0.26), sexual optimism (β = 0.20, marginal), and sexual problem self-blame (β = −0.16, marginal) were the only substantive multiple regression predictors, accounting for about 22% of the variance in sexual satisfaction (adjusted *R*^2^ reported). Relationship status was not a multiple regression predictor (β = 0.02). Standardized betas for all variables are presented in Table 4.

#### 3.4.2. Correlations of Relationship Satisfaction

For those in a relationship, satisfaction with sexual functioning and relationship satisfaction were slightly-to-moderately correlated (*r* = 0.36, *N* = 95). Table 4 presents correlations of satisfaction with one’s romantic relationship with sexual self-concept, comfort with sexuality, and past sexual behavior for those who were involved in more and less serious romantic relationships. By definition, single people are not in a relationship, so these participants were not included in relationship satisfaction analyses.

The pattern of correlations were quite different, contingent upon relationship type; generally, those in less serious relationships expressed more relationship dissatisfaction when they had favorable sexual self-concepts. In contrast, those in more serious relationships tended to express more relationship satisfaction when they had favorable sexual self-concepts. Regardless of the differential pattern, both of these relationships were modest at best (i.e., average *r*_more serious_ = 0.31 and *r*_less serious_ = 0.20). Partial correlations where relationship status (more serious or less serious) was controlled attenuated the relationships between sexual self-concept and relationship satisfaction.

In terms of comfort with sexuality, those in less serious relationships tended to express more relationship dissatisfaction when they were more erotophilic. In contrast, those in more serious relationships tended to report more satisfaction with their relationship when they were more comfortable with sexual behavior and were more comfortable with children’s sexual curiosity. There were no consistent patterns of relationship satisfaction correlations with past sexual behavior; the majority were not significant.

A multiple regression analysis was conducted where relationship satisfaction was the dependent variable and the sexual self-concept variables, comfort with sexuality variables, and sexual experience variable as well as relationship status (i.e., more or less serious) were predictor variables. The equation was significant (*F*(12, 65) = 4.17, *p* < 0.001, *R* = 0.66) where relationship status (β = 0.60) and erotophobia–erotophilia (β = −0.34) were significant predictors (Table 4); the equation accounted for 33% of the variance in relationship satisfaction (adjusted *R*^2^ reported). In contrast to satisfaction with sexual functioning, no sexual self-concept variable was a significant predictor of relationship satisfaction.

## 4. Discussion

The main research question of this investigation was: Are there differences in sexual selfhood as a function of relationship status? The pithy answer is a qualified yes. On some measures of sexual self-concept (e.g., sexual self-schema, sexual self-efficacy), participants in serious romantic relationships had more favorable self-characterizations than those who were not in relationships. Those not in relationships were less erotophilic and more uncomfortable with sexual behavior compared to those in relationships, although this was only multivariately marginally significant and effect sizes were small. Participants in relationships had more sexual experience relative to those who were not in a relationship.

Some scales from Snell’s Multidimensional Sexual Self-Concept instrument [39] demonstrated relationship-based differences; the strongest effect was for sexual self-schema followed by sexual self-efficacy, sexual consciousness, and sexual optimism. These findings are consistent with research suggesting that individuals in a romantic relationship are likely to have more enhanced aspects of sexual self-concept [22,31,36]. However, Kislev [36] found that married individuals had lower levels of sexual self-esteem and sexual assertiveness compared to those classified in other relationship types—most notably, those who were single. This contrasts with the current sexual self-concept findings but might be a result of research design differences; the current study did not assess sexual self-esteem nor sexual assertiveness (called communication by Kislev but akin to Snell and Kilimnik’s assertiveness [39]). Further, the current study specifically solicited emerging adults (first-year university students; typically aged 19–20 years) who would have temporally limited “serious relationships” compared with Kislev’s sample, where participants were restricted to those over 30 years of age (average age 39 years). Further, the current definition of relationship status was less refined in that it entailed three relationship levels—although consistent with a similar three-group romance paradigm for youth used by Kindelberger et al. [46]—compared to Kislev’s seven relationships levels. Kislev’s typology represented a more nuanced relational labeling of people who would be at different ages and stages in their relationship lives. This illustrates the importance of considering who the participants are in a study and the type of cohort to which the findings apply as well as operational definitions of key constructs such as sexual selfhood and relationship status.

The current results also indicated greater comfort with sexuality for those in romantic relationships. This discomfort could be a result of experience with sexuality and relationships. Some of the literature supports the influence of sexual comfort in areas other than intimate relationships; Shindel et al. [47] found that medical students with less sexual experience (i.e., virginity, fewer sexual partners, lower sexual behavior frequency) expressed greater discomfort with addressing patients’ sexual health. Further, Davies and Windle [35] found that individuals engaged in more disclosure with friends—that is, general intimacy—when involved in a relationship versus when single. It is possible that being in a romantic relationship influences many aspects of the self, behavior, and identity development.

The current findings are consistent with Agnew et al.’s [48] discussion of cognitive interdependence in close relationships—how individuals incorporate their romantic partner into their conceptualizations of themselves—and with Burris and Rempel’s [49] theory that self-concept changes when it is expanded to include a romantic partner. One function of relationships is to meet a host of needs of the romantic partners. This need fulfillment helps lead to further interdependence, resulting in a sense of self that incorporates the other (i.e., a movement from “me” to “we”). Cognitive interdependence increases with relationship commitment and intimacy. It follows logically that having sexual needs met, and meeting the sexual needs of the other, could have an effect on the cognitive ways in which one thinks about their sexual self. This may explain why we see more positive sexual self-concept differences in those in relationships, particularly more serious relationships.

While the findings of this study provide some evidence that individuals in relationships differ from those not in a relationship in ways that are consistent with cognitive interdependence theory, the causal aspect of the theory, that is, whether these changes result from entering a romantic relationship, cannot be addressed adequately in a cross-sectional design study. Due to the correlational nature of the current study, it is impossible to conclude that relationship status differences are responsible for differences in sexual self-concept. The causal path from relationship status to sexual selfhood could be addressed more strongly using a longitudinal study akin to Kindelberger et al. [46]. They conducted a time-panel analysis of late adolescent relationship status and found that general identity development processes were influenced by being in a serious romantic relationship but found no such effects for more casual relationships. Whether the Kindleberger et al. findings [46] would be replicated with specific sexual identity measures, such as sexual self-concept [39] or sexual identity development commitment [14], is worthy of further investigation.

A secondary question addressed in this study was whether relationship status, sexual self-concept, comfort, and sexual experience predicted satisfaction with sexual functioning as well as relationship satisfaction. Sexual functioning satisfaction was not predicted by relationship status, but most of the scales from the Multidimensional Sexual Self-Concept instrument were related significantly at a bivariate level. In terms of multiple regression, sexual consciousness and sexual self-schema were significant predictors, whereas relationship status was not. Regarding bivariate relationships of the sexual selfhood variables and relationship satisfaction of those participants in a relationship, some comfort with sexuality variables and a few sexual self-concept variables (e.g., self-schema, motivation to avoid risk sex) were related to relationship satisfaction at the zero-order level. Excluding single participants, being in a more serious relationship and erotophobia–erotophilia were multiple regression predictors of relationship satisfaction. These findings need to be qualified by the fact that the satisfaction outcome variables were single items.

The findings regarding sexual satisfaction being predicted by sexual self-concept variables is consistent with Antičević et al. [22] as well as Kislev [36]; Muise et al. [50] also found several aspects of sexual self-concept loaded with sexual satisfaction as a latent construct they called sexual well-being. In a study of male Chinese youths, To et al. [51] found that sexual self-concept dimensions of consciousness and self-esteem predicted sexual satisfaction at a bivariate level. In a sample of high school couples, Couture et al. [52] found that comfort with sexuality was modestly correlated with sexual satisfaction, similar to the current investigation. Relationship and sexual functioning satisfaction exhibited a modest association in this study, which is consistent with other research [38]. Arcos-Romero and Sierra [53] found that erotophobia–erotophilia did not correlate well with relationship satisfaction in a sample of adults who were in 6+-month relationships, whereas the current study found erotophobia-erotophilia to be the only predictor of relationship satisfaction. Similar to Kislev, the current study assessed sexual satisfaction using a single item, so the results should be considered with caution as there are better measures of sexual satisfaction [54].

The findings of the current study could have theoretical implications. Knowing that romantic relationships have the potential to impact sexual self-concept and comfort with sexuality, future research could examine sexual selfhood constructs and relationship status as theoretical determinants of relational satisfaction and psychological well-being, perhaps mediated by sexual satisfaction. There may also be practical implications of this study. Clinicians may want to highlight or address these psychosexual areas for couples who are struggling to communicate or connect as sexual self-concept and sexual-comfort have been found to relate to psychological well-being [55] as well as being laudable outcomes for which to strive in and of themselves [56]. However, sexual selfhood variables seem to differ across samples and potentially across age cohorts. For example, comparing some of the same measures in this study to other studies of emerging adults suggested that the current sample had higher levels of both positive and negative sexual self-concept domains (i.e., sexual consciousness, sexual problem self-blame, sexual self-efficacy [51,57]). Further investigation from a clinical perspective is warranted.

## 5. Conclusions

In this sample of emerging adults, sexual selfhood differed in many respects as a function of relationship status. Sexual self-concept, comfort with sexuality, and sexual experience all demonstrated some modest differences as a function of relationship status. Generally, those in a romantic relationship—particularly a more serious relationship—tended to have more favorable sexual self-concepts, greater comfort with sexuality, and greater sexual experience. Further, these components of the psychosexual self were somewhat predictive of sexual and relationship satisfaction. These findings conceptually replicate the findings of such researchers as Antičević et al. [22] and Kislev [36]. However, when considering the impact of relationship status on the sexual self, it is extremely important to consider developmental life span perspectives.

## Figures and Tables

**Table 1:** Correlations between the sexual self-concept scales, comfort with sexuality scales, and past sexual behaviors.

		Bivariate Correlations					
Self-Concept (*n* = 178–179)	$\overset{-}{x}$/SD	Self-Efficacy	Conscious-ness	Optimism	Problem Self-Blame	Power/Other Sexual Control	Motivation to Avoid Risky Sex
Self-Schema	3.91/0.92	0.64 ***	0.53 ***	0.43 ***	0.10	−0.21 **	0.18 *
Self-Efficacy	3.78/0.90	1	0.74 ***	0.34 ***	0.30 ***	−0.18 *	0.16 *
Consciousness	4.01/0.82		1	0.19 *	0.14 ^marg^	−0.19 *	0.19 *
Optimism				1	0.12	−0.45 ***	0.34 ***
Problem Self-Blame	3.16/0.97				1	0.19 *	0.04
Power/Other Sexual Control	1.98/1.01					1	−0.28 ***
Motivation to Avoid Risky Sex	4.51/0.63						
Comfort with Sexuality (*n* = 178–180)							
	$\overset{-}{x}$/SD	Comfort with Sexual Behavior		Erotophobia–Erotophilia		Attitudes toward Sexual Curiosity in Children	
Comfort with Sexual Behavior	3.95/98		1		0.62 ***		0.47 ***
Erotophobia–Erotophilia	4.57/1.08				1		0.57 ***
Attitudes toward Sexual Curiosity in Children	4.24/0.81						
Past Sexual Behaviors (*n* = 148–168)							
	$\overset{-}{x}$/SD	# of other-sex partners	# of one-night stands	# of same-sex partners	Sexual Experience		
# of other-sex partners	2.63/3.76	1	0.66 ***	0.12	0.49 ***		
# of one-night stands	0.56/1.42		1	0.39 ***	0.23 ***		
# of same-sex partners	0.29/1.03			1	0.00		

Notes: *** *p* < 0.0001; ** *p <* 0.01; * *p* < 0.05; ^marg^ *p* > 0.05 and *p* < 0.10; # = number. Self-concept responses ranged from 1 to 5. A score of 4 on Comfort with Sexual Behavior corresponded with “All right if you are deeply in love with the person/dating seriously”. Attitudes toward sexual curiosity in children and erotophobia—erotophilia responses ranged from 1 to 7; a score of 7 was most liberal.

**Table 2:** Differences in Sexual Variables as a Function of Relationship Status: Descriptive Statistics and Univariate Tests.

Dependent Variable	$\mathrm{More}\;\mathrm{Serious}\;\mathrm{Relationship}\;(\overset{-}{x}$/SD) *N* = 47–53	Less Serious Relationship $(\overset{-}{x}$/SD) *N* = 28–37	Single $(\overset{-}{x}$/SD) *N* = 71–88	*F* *(df)*	η_p_^2^
Sexual Self-Concept				(2, 174)	
Self-Schema	4.28/0.78 _a_	4.15/0.78 _a_	3.59/0.95 _b_	12.35 ***	0.12
Self-Efficacy	4.12/0.77 _a_	3.90/0.86 _a_	3.53/0.92 _b_	8.15 ***	0.09
Consciousness	4.23/0.74 _a_	4.18/0.70 _a_	3.81/0.86 _b_	5.68 **	0.06
Optimism	4.11/0.62 _a_	3.72/0.56 _b_	3.78/0.75 _b_	4.99 **	0.05
Problem Self-Blame	3.02/0.94	3.00/0.85	3.32/1.02	2.28	0.03
Power/Other Sexual Control	1.88/0.83	1.91/0.79	2.07/1.01	0.80	0.01
Motivation to Avoid Risky Sex	4.48/0.62	4.55/0.49	4.51/0.70	0.11	0.00
Comfort with Sexuality				(2, 174)	
Comfort with Sexual Behavior	4.23/0.72 _a_	4.05/0.89 _ab_	3.74/1.09 _b_	4.57 *	0.05
Erotophobia–Erotophilia	4.82/1.03	4.78/0.95	4.33/1.07	4.45 *	0.05
Attitudes toward Sexual Curiosity in Children	4.37/0.80	4.24/0.90	4.16/0.79	1.07	0.01
Past Sexual Behavior				(2, 143)	
Sexual Experience	2.66/0.60 _a_	2.21/0.83 _b_	1.73/0.83 _c_	21.14 ***	0.23
# of other-sex partners	3.32/3.48 _ab_	3.64/4.47 _a_	1.80/3.17 _b_	3.95 *	0.05
# of one-night stands	0.66/1.71	0.75/1.21	0.42/1.26	0.71	0.01
# of same-sex partners	0.13/0.34	0.39/1.26	0.39/1.33	0.95	0.01

Notes: Where significant univariate effects are reported, means with different subscripts _abc_ differ significantly from each other using Student–Newman–Keuls post hoc analysis. Range of scores for sexual self-concept variables was 1–5, while Comfort with Sexual Behavior and Attitudes toward Sexual Curiosity in Children was 1–6. Erotophobia–erotophilia could range from 1 to 7. Levene’s test of equality of variance was non-significant for all except Comfort with Sexual Behavior and the Sexual Experience variables. For both univariate tests, greater variance was associated with the group with the largest *n*, thus overestimating the *p* value [45]. *** *p* < 0.0001; ** *p* < 0.005; * *p* < 0.05; # = number.

**Table 3:** Structure matrix loadings from discriminant function analyses.

Dependent Variable	Function 1 *r*	Function 2 *r*
Sexual Self-Concept		
Self-Schema	0.77	−0.16
Self-Efficacy	0.63	0.07
Consciousness	0.52	−0.16
Optimism	0.36	0.68
Problem Self-Blame	−0.32	0.19
Power/Other Sexual Control	−0.20	0.05
Motivation to Avoid Risky Sex	−0.02	−0.14
Comfort with Sexuality		
Comfort with Sexual Behavior	0.88	0.45
Erotophobia–Erotophilia	0.88	−0.23
Attitudes toward Sexual Curiosity in Children	0.40	0.50
Past Sexual Behavior		
Sexual Experience	0.97	0.16
Number of other-sex partners	0.35	0.90
Number of one-night stands	0.14	0.43
Number of same-sex partners	−0.18	0.32

**Table 4:** Relations of Sexual Functioning and Relationship Satisfaction with Sexual Self-Concept, Comfort with Sexuality, and Past Sexual Behavior.

	Satisfaction with Sexual Functioning						Relationship Satisfaction		
Sexual Self-Concept	More Serious Relationship (*n* = 52–53)	Less Serious Relationship (*n* = 36)	Single (*n* = 79)	Partial *r* (*n* = 167–169)	β	More Serious Relationship (*n* = 52–53)	Less Serious Relationship (*n* = 36)	Partial *r* (*n* = 77–84)	β
Self-Schema	0.33 *	0.11	0.45 ***	0.34 ***	0.26 *	0.36 **	−0.32 ^marg^	−0.02	0.06
Self-Efficacy	0.30 *	0.15	0.29 ***	0.27 ***	−0.11	0.16	−0.31 ^marg^	−0.12	−0.12
Consciousness	0.34 *	0.24	0.22 ^marg^	0.26 ***	0.33 *	0.19	−0.20	−0.03	0.18
Optimism	0.45 **	0.10	0.35 **	0.33 ***	0.20 ^marg^	0.41 **	−0.20	0.09	0.03
Problem Self-Blame	−0.24 ^marg^	0.20	−0.04	−0.05	−0.16 ^marg^	−0.11	−0.26	−0.17	−0.04
Power/Other Sexual Control	−0.22	0.03	−0.26 *	−0.19 **	0.01	−0.34 *	0.02	−0.14	−0.07
Motivation to Avoid Risky Sex	0.41 **	−0.21	0.12	0.16 *	−0.05	0.55 ***	−0.07	0.22 *	0.10
Comfort with Sexuality									
Erotophobia–Erotophilia	0.11	−0.10	0.01	0.01	−0.10	0.08	−0.43 *	−0.20 ^marg^	−0.34 *
Comfort with Sexual Behavior	0.23	−0.12	0.05	0.05	0.01	0.34 *	−0.22	−0.01	0.06
Attitudes toward Sexual Curiosity in Children	0.21	−0.02	0.01	0.06	0.03	0.31 *	0.11	0.17	0.13
Past Sexual Behavior	*n* = 45–52	*n* = 21–30	*n* = 21–76	*n* = 87–160		*n* = 45–52	*n* = 21–28		
Sexual Experience	0.05	0.13	0.28 *	0.17 *	0.06	0.09	0.12	−0.07	0.02
# of other-sex partners	0.08	0.01	0.25 *	0.14	na	0.10	0.48 *	−0.21 ^marg^	na
# of one-night stands	0.01	−0.07	0.16	0.06	na	0.06	−0.31	−0.10	na
# of same-sex partners	−0.25 ^marg^	−0.06	0.00	−0.04	na	−0.20	−0.22	−0.10	na
Age of 1st Intercourse	−0.12	−0.16	0.05	−0.09	na	0.09	−0.22	−0.03	na
Relationship Status					0.02				0.60 ***

Notes:* *p* < 0.05; ** *p* < 0.01; *** *p* < 0.0001; ^marg^ *p* > 0.05 and *p* < 0.10; # = number; na = not applicable. Partial *r* represents the relationship between the two variables controlling for relationship status. β is the standardized beta in the multiple regression. For both multiple regression analyses, multicollinearity was not a problem based on VIF and tolerance statistics; assumptions of independent errors were met based on Durbin–Watson statistics; and 11 and 4 outliers were omitted for sexual functioning and relationship satisfaction, respectively, based on Mahalanobis distance and Centered Leverage values. All Cook’s distances were within acceptable parameters. Assumptions of linearity, normality of residuals, and homoscedasticity were met [45].

## Data Availability

Participants of this study were not asked for permission for their data to be shared publicly, so supporting data are not available.
